# Study on the Effect of Quinoa Saponins on Human Colon Cancer HT‐29 Cells

**DOI:** 10.1002/fsn3.4669

**Published:** 2024-12-19

**Authors:** Haijun Shang, Jinwei Sun, Zhi Zheng, Shujun Sun, Xiaoming Yan

**Affiliations:** ^1^ College of Food and Biological Engineering Hefei University of Technology Hefei China; ^2^ Anhui Business and Technology College Hefei China; ^3^ Fuyang Normal University Fuyang China

**Keywords:** apoptosis, autophagy, cell cycle, colon cancer, migration, quinoa saponins

## Abstract

Quinoa saponins can inhibit the survival of specific cancer cells. However, there is still a lack of systematic research on the effects of quinoa saponins on colon cancer cells. This experiment confirmed that quinoa saponins prevented human colon cancer HT‐29 cells from growing in vitro. The MTT experiment revealed that quinoa saponins significantly decreased the proliferative vitality of HT‐29 cells. In comparison to the control group, the proportion of cell number in the G0/G1 phase increased by 22.97% and the rate of apoptosis increased by 22.55% after treating cells with quinoa saponins (40 μg/mL). By regulating the expression of Cyclin D1 and p21, it caused the cell cycle to be blocked in the G0/G1 phase. It also promoted the expression of Caspase3 and Bax while suppressing the expression of Bcl‐2, which led to the apoptosis of HT‐29 cells. In addition, quinoa saponins caused cells to undergo autophagy by upregulating the expression of LC‐3II and Beclin1, while the addition of autophagy inhibitors significantly reduced the inhibitory effect on cell proliferation. Finally, the migration of HT‐29 cells was also inhibited by quinoa saponins. After treating cells with quinoa saponins (40 μg/mL), compared with that in the control group, the wound healing rate of cells decreased by 38.21% and the migration ability decreased by 69.48%. The potential mechanism could be connected to increasing E‐cadherin expression while decreasing N‐cadherin expression. Importantly, all of these changes induced by quinoa saponins were dose dependent. Overall, these findings give a scientific basis for the anticancer mechanism of quinoa saponins.

## Graphical abstract

Isolation of high‐purity monomers from crude saponins of quinoa and identification of their structures. Quinoa saponins inhibit the growth of human colon cancer cells HT‐29 in vitro. Quinoa saponins affect the survival of HT‐29 colon cancer cells by regulating the expression of key proteins.

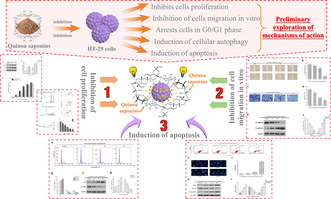

## 1 Introduction

Colon cancer has the fourth highest incidence rate of all cancers, and roughly 550,000 people die from it each year (Jayasinghe et al. 2023). The pathogenesis of colon cancer is very complex, with genetic mutations; the environment or poor dietary habits may lead to colon cancer. Currently, surgery, chemotherapy, and radiation therapy are the primary methods used to treat colon cancer (Morii et al. 2021). The number of deaths is rising despite patients being treated through these methods (Dey et al. 2023). This is due to the problems of complications, high toxicity and side effects of radiotherapy, which have not yet been effectively addressed (Eyraud 2015). As a result, more effective therapeutic targets and strategies are needed. According to the world epidemiological monitoring and prognostic data, the incidence of colon cancer tends to be younger, which is greatly related to the irrational dietary habits of young people (Siegel et al. 2020; Wang et al. 2017).

Diet has a significant role in preventing cancer (Grosso et al. 2017). Recent findings show that regular consumption of vegetables and fruits can reduce the incidence of cancer (Li, Liu, et al. 2015). This is due to the extraction of saponins, flavonoids, phenolic compounds and active substances from them, which have a powerful toxic effect on cancer cells (Gupta, Ahuja, and Gupta 2022). Especially saponins have great potential in the field of anticancer, and numerous experiments have found that saponins have inhibitory effects on colon cancer cells. Zhang et al. (2022) found that 20(S)‐ginsenoside Rh2 had an inhibitory effect on the growth of colon cancer cells by suppressing the Axl signaling pathway. Yao et al. (2020) found that PP9 (a steroidal saponin) significantly affected the survival of colon cancer cells, with a more pronounced effect than 5‐Fu. Therefore, saponins provide novel approaches and strategies for treating colon cancer.

Quinoa (*Chinopodium quinoa* Willd.) belongs to the annual quinoa family, whose agricultural history dates back to about 5000–7000 years ago (Contreras‐Jiménez, Torres‐Vargas, and Rodríguez‐García 2019; Song et al. 2024). It is mainly planted in high‐altitude areas of South America (Sezgin and Sanlier 2019) and has the characteristics of cold tolerance, salt tolerance, and drought tolerance (Vilcacundo and Hernández‐Ledesma 2016). Quinoa is the “golden grain” in agriculture. It is rich in nutrients such as protein, dietary fiber, vitamins, lipids, and minerals (Li et al. 2024; Saeid et al. 2024), and secondary metabolites, such as saponins, phenols, and flavonoids (Ren et al. 2023). Regular consumption of quinoa products can prevent cancer, lower blood sugar, lose weight, and lower blood lipids (Lin et al. 2021; Ng and Wang 2021). Chemical analysis of quinoa showed that saponins were the main bioactive components in quinoa. Quinoa saponins belong to plant triterpenoid saponins (Yang et al. 2024), which consist of one or more sugar chains linked to aglycones (Taco et al. 2022). The outer coat of quinoa is rich in saponins, which are often discarded due to their bitter taste (Oulkhir et al. 2023). Quinoa saponins are considered to be the main beneficial phytochemicals for health, with pharmacological activities such as antioxidant, antibacterial, antitumor, and immune regulation (Abdelshafy, Rashwan, and Osman 2024; Starzyńska‐Janiszewska et al. 2023). Especially in anticancer, quinoa saponins have significant antitumor effects. Kuljanabhagavad et al. (2008) isolated and characterized 20 triterpenoid saponins from different parts of quinoa, which were found to have toxic effects on cervical adenocarcinoma HeLa cells. Albadri (2020) found that quinoa saponins had an inhibitory effect on the survival of breast cancer cells. Resveratrol glycosides in quinoa have toxic effects on colon cancer cells, but there is a lack of systematic research data on whether quinoa saponins have inhibitory effects on colon cancer cells.

In order to further explore the anticancer activity of quinoa saponins, this experiment mainly focused on the effect of quinoa saponins on colon cancer cells. The quinoa saponins were extracted and isolated from the seed coat of quinoa. In vitro experiments were conducted to examine the effects of quinoa saponins on the migration, cell cycle, apoptosis, and autophagy of HT‐29 cells. In addition, the expression of relevant proteins was detected by Western blotting to explore possible‐related mechanisms.

## 2 Materials and Methods

### 2.1 Main Reagents and Materials

The seed coat of quinoa was obtained from Qingli No. 2 cultivated by Qinghai Academy of Agricultural Sciences, with white grains and a plant height of 170 cm, and the seed coat was dried to a constant weight without rotting (Xining, China). Human colon cancer HT‐29 cells and McCoy's 5A medium were obtained from Wuhan Prosai Life Technology Co. Ltd. (Wuhan, China); FBS, penicillin/streptomycin mixture, MTT, and Hoechst 33342 staining solution were obtained from Sangong Bioengineering Co. Ltd. (Shanghai, China); annexin V‐FITC/PI apoptosis detection kit was obtained from Jiangsu KeyGEN Biotechnology Co. Ltd. (Nanjing, China); DMSO was obtained from Sigma (St. Louis, Missouri, USA); 3‐MA and PI staining solution were obtained from Shanghai Yuanye Biotechnology Co. Ltd. (Shanghai, China); and WB antibodies were obtained from Wuhan Servicebio Technology Co. Ltd. (Wuhan, China).

### 2.2 Extraction and Isolation of Quinoa Saponins

**FIGURE 1:**
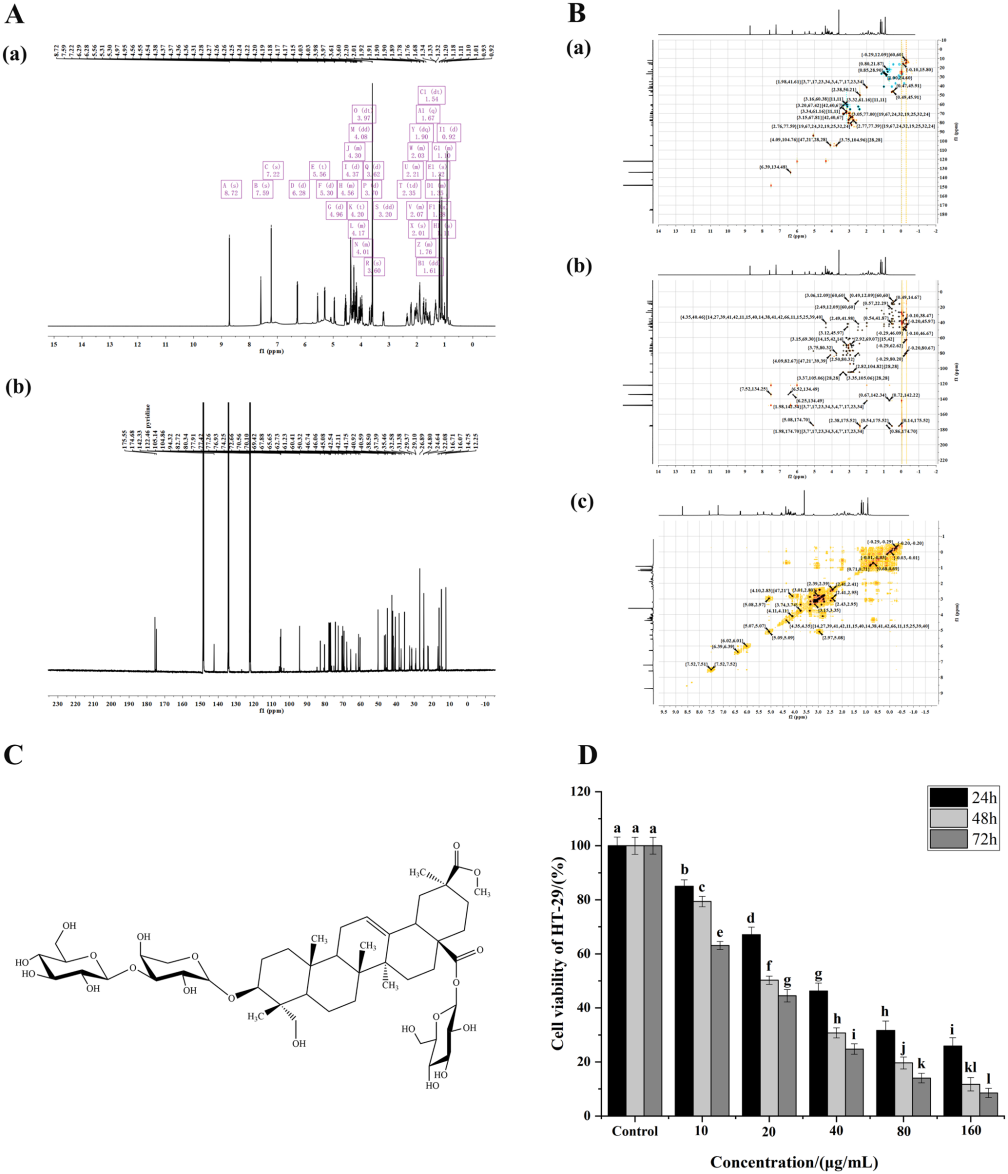
Quinoa saponins inhibited cell proliferation. (A) One‐dimensional nuclear magnetogram: (a) and (b) are the hydrogen spectrogram and carbon spectrogram, respectively. (B) Two‐dimensional nuclear magnetogram: (a), (b), and (c) are the HSQC, HMBC, and H‐HCOSY spectrogram, respectively. (C) The chemical structure of quinoa saponins. (D) MTT assay was used to detect the proliferation of HT‐29 cells treated with different concentrations of quinoa saponins for 24, 48, and 72 h. Different letters indicate significant differences, *p* < 0.05.

The method of extraction of quinoa saponins was slightly modified from the previously reported method (Madl et al. 2006). Quinoa seed coat powder was treated with 1.5 L of water/ethanol (3:7). The treated sample was stirred at 50°C and then filtered in vacuum and concentrated in a rotary evaporator. The saponins were extracted with saturated n‐butanol for three times. The n‐butanol layers were merged and evaporated by rotary evaporation to obtain the crude saponins. Following absorption by the macroporous resin D101, 70% ethanol was used to elute the crude saponins. The eluate was separated by semi‐preparative liquid chromatography after rotary evaporation and freeze‐drying (Waters 2535, Waters, MA, USA). The chromatographic column was a C_18_ column (Sepax GP‐C_18_, 250 mm × 10.0 mm, 5 μm, Sepax Technologies Inc., Suzhou, China). The mobile phases were methanol (A) and water (B) at a flow rate of 5 mL/min, and the detection wavelength was 210 nm. The gradient conditions for elution were as follows: 0 to 5 min, 10% to 70% A; 5 to 20 min, 70% to 85% A; 20 to 25 min, 85% to 90% A; 25 to 26 min, 90% to 10% A; and 26 to 30 min, 10% A. Quinoa saponins were obtained (white amorphous powder; yield: 285 mg). After NMR identification (Figure 1A,B), it was determined that the structure of the isolated compound was as in Figure 1C.

### 2.3 Cell Viability Assay

As mentioned before, MTT was utilized to assay cell viability (Gomez de Cedron et al. 2020). The cells were treated with quinoa saponin solutions (10, 20, 40, 80, and 160 μg/mL) for 24, 48, and 72 at 37°C in a 5% CO_2_ incubator. Following incubation, MTT (20 μL) was applied to each well and incubated for 4 h. After forming formazan crystals, DMSO (200 μL) was applied to each well and shaken for 5 min. A microplate analyzer was used to test the cells' absorbance at 540 nm (Thermo Multiskan Go 1510, Thermo Fisher Scientific, MA, USA).

### 2.4 The Cell Cycle Analysis

As mentioned before, the cell cycle distribution was investigated using flow cytometry (Xu et al. 2020). The cells were treated with quinoa saponins for 24 h. Following that, the cells were fixed at 4°C for the night after being resuspended in 70% ice ethanol. Then the cells were treated with staining reagent (50 μg/mL PI, containing RNase). The distribution of cell cycles was promptly examined using flow cytometry (Beckman CytoFLEX, Beckman Coulter Inc., CA, USA).

### 2.5 Cell Morphological Assessment

As mentioned before, Hoechst 33342 dye was utilized to assess the cell morphology (Yao et al. 2020). The cells were treated with quinoa saponins for 24 h. Following two PBS rinses, the cells were treated with Hoechst 33342 (10 μg/mL) for 15 min and then fixed with 4% paraformaldehyde for 30 min. A fluorescent microscope was used to observe the cells (Nikon 80i, Japan).

### 2.6 The Cell Apoptosis Analysis

As mentioned before, a FITC‐labeled Annexin V/PI was utilized to measure apoptosis‐mediated tumor cell death (Lai et al. 2022). The cells were treated with quinoa saponins for 24 h. 1 × Binding Buffer was added. Following the addition of AV and PI, the mixture was incubated in the dark for 15 min. A flow cytometer was used to detect the apoptosis (Beckman CytoFLEX, Beckman Coulter Inc., CA, USA).

### 2.7 Effect of Autophagy Inhibitors on Cell Proliferation

As mentioned before, 3‐MA was utilized to examine how autophagy affects the proliferation of cancer cells ( Li, Yang, et al. 2015). The cells were pretreated with 5 mM 3‐MA for 2 h and then treated with quinoa saponins for 24 h. After adding 20 μL MTT to each well, HT‐29 cells continued to be incubated for 4 h. DMSO (200 μL) was added and shaken slowly. At 540 nm, the cells' absorbance was measured.

### 2.8 Wound Healing and Transwell Migration Assay

As mentioned before, the cell wound healing was determined by scratching the cells (Ji et al. 2021). When the cell density reached 90%, the cells were scratched. The cells were treated with quinoa saponins for 24 h and observed under an inverted microscope. As mentioned before, the transwell was used to evaluate the capacity for cell migration (Song et al. 2015). Following treatment with quinoa saponins, the cells were suspended in serum‐free media and injected at a density of 5 × 10^4^ wells in Transwell chambers. The lower layer of Transwells was supplemented with 600 μL of serum‐free medium. After 24 h, the migrated cells were fixed with 4% paraformaldehyde and stained with 0.1% crystal violet, and then washed with PBS. The transwell chamber was photographed and examined under a microscope. Glacial acetic acid was added and shaken for 5 min, and the absorbance was measured at 570 nm.

### 2.9 Western Blot Analysis

As mentioned before, cellular proteins were analyzed by Western blotting (Chang et al. 2021). After treatment of cells with quinoa saponins, total protein was extracted by adding RIPA cell lysate and collected by centrifugation. SDS‐PAGE electrophoresis was used to separate the proteins. The proteins were transferred to a PVDF membrane following electrophoresis. The strips were removed after being closed with 5% skimmed milk for 1 h at 37°C. The primary antibody was incubated at 4°C for the entire night, the membrane was washed with TBST, and the secondary antibody was applied and incubated for 1 h. After washing the membrane with TBST, it was developed with ECL.

### 2.10 Statistical Analysis

SPSS 26.0 was utilized for the statistical analysis. The measurements were expressed as mean ± standard deviation, and the results were analyzed using ANOVA or *t*‐test.

## 3 Results

### 3.1 Quinoa Saponins Inhibited HT‐29 Cells' Proliferation

The MTT assay is used to determine cell vitality by using active metabolites in the cells to reduce MTT to purple impurities and by detecting the absorbance of these purple impurities. As shown in Figure 1D, the proliferative vitality of HT‐29 cells was significantly decreased after treatment with quinoa saponins. With the increase in the concentration and treatment time of quinoa saponins, the inhibitory effect on cell growth increased significantly. The proliferative vitality of HT‐29 cells was significantly reduced after treatment with quinoa saponins (40 μg/mL). The survival rates of cancer cells treated with quinoa saponins (160 μg/mL) for 24, 48, and 72 h were 25.89%, 11.75%, and 8.53%, respectively. The IC_50_ values were 24.11, 18.87, and 14.52 μg/mL, respectively. The above results suggested that quinoa saponins inhibited the growth of HT‐29 cells in a time‐dependent and dose‐dependent manner. This is consistent with the results of Wang and Li who studied the inhibitory effects of plant saponins on colon cancer cells (Wang et al. 2018; Li et al. 2018). The present study indicates that cell growth viability is affected by cell cycle distribution, apoptosis, and autophagy to some extent.

### 3.2 Quinoa Saponins Arrested HT‐29 Cells in G0/G1 Phase

**FIGURE 2:**
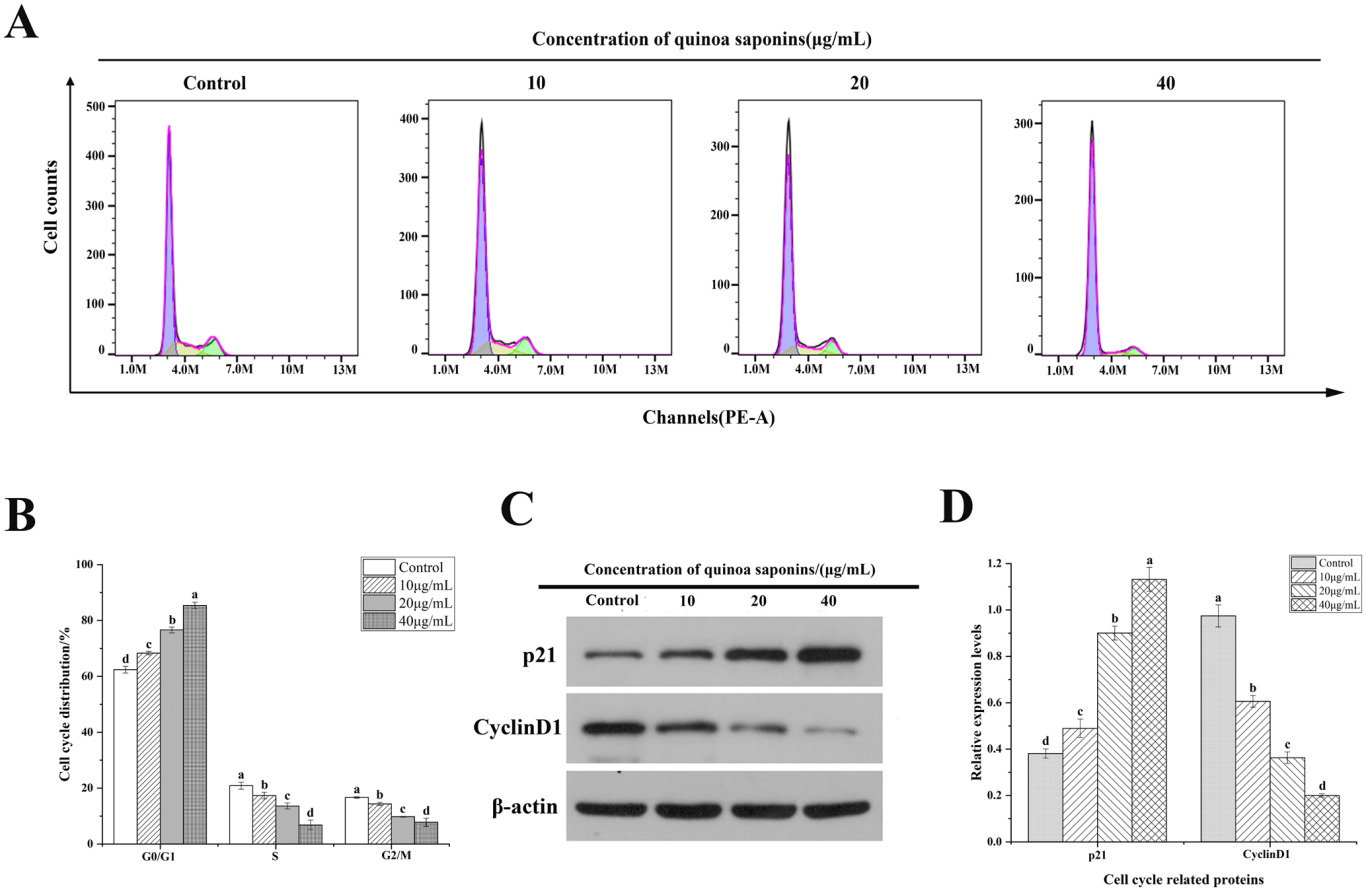
Quinoa saponin‐induced G_0_/G_1_ cell cycle arrest in HT‐29 cells. (A) and (B) The cycle distribution of HT‐29 cells treated with different concentrations of quinoa saponins was detected by flow cytometry. (C) and (D) The expression levels of cyclin D1 and p21 were detected by Western blot. Different letters indicate significant differences, *p* < 0.05.

Cell cycle arrest is an important factor in preventing cell proliferation. As shown in Figure 2A,B, as the concentration of quinoa saponins increased, the proportion of G0/G1‐phase cells rose significantly, with a corresponding decrease in the proportion of S‐phase and G2/M‐phase cells. In particular, the effect on the cycle was most pronounced when cells were treated with quinoa saponins (40 μg/mL), with a 22.97% increase in the G0/G1‐phase cells and a 14.07% decrease in the S‐phase cells compared with those of the control group. The research results indicated that quinoa saponins blocked HT‐29 cells in the G0/G1 phase. Gao et al. (2018) discovered that ophiopogonin B treatment could inhibit the growth of HT‐29 cells by blocking them in the G0/G1 phase, which is in line with the results of this experiment. In contrast, Yao et al. (2020) discovered that the saponin PP9 effectively induced HT‐29 cells to block in the G2/M phase, which was different from the cell cycle blocked in this experiment. This may be due to the fact that saponins affect the distribution of the cell cycle by regulating the expression of different proteins in the cells, thereby affecting the growth of cancer cells.

In order to further investigate the mechanism of quinoa saponins on cell cycle, the effect of quinoa saponins on the expression of proteins related to the G0/G1 phase of the cell cycle was examined by Western blotting. During the G0/G1 phase, the cell arrest may result from low Cyclin D1 expression and high p21 expression (Lim and Kaldis 2013). As shown in Figure 2C,D, the expression of p21 was significantly increased and the expression of Cyclin D1 was decreased after the cells were treated with quinoa saponins. This is consistent with Lee's findings on the effect of CME saponins on cell cycle proteins in HCT116 cells. CME significantly reduced Cyclin D1 expression, resulting in cell cycle arrest in the G0/G1 phase, which inhibited the growth of cancer cells (Lee et al. 2015). Therefore, it can be inferred that quinoa saponins affect cell growth by regulating key proteins that arrest HT‐29 cells in the G0/G1 phase.

### 3.3 Quinoa Saponin‐Induced Apoptosis of HT‐29 Cells

**FIGURE 3:**
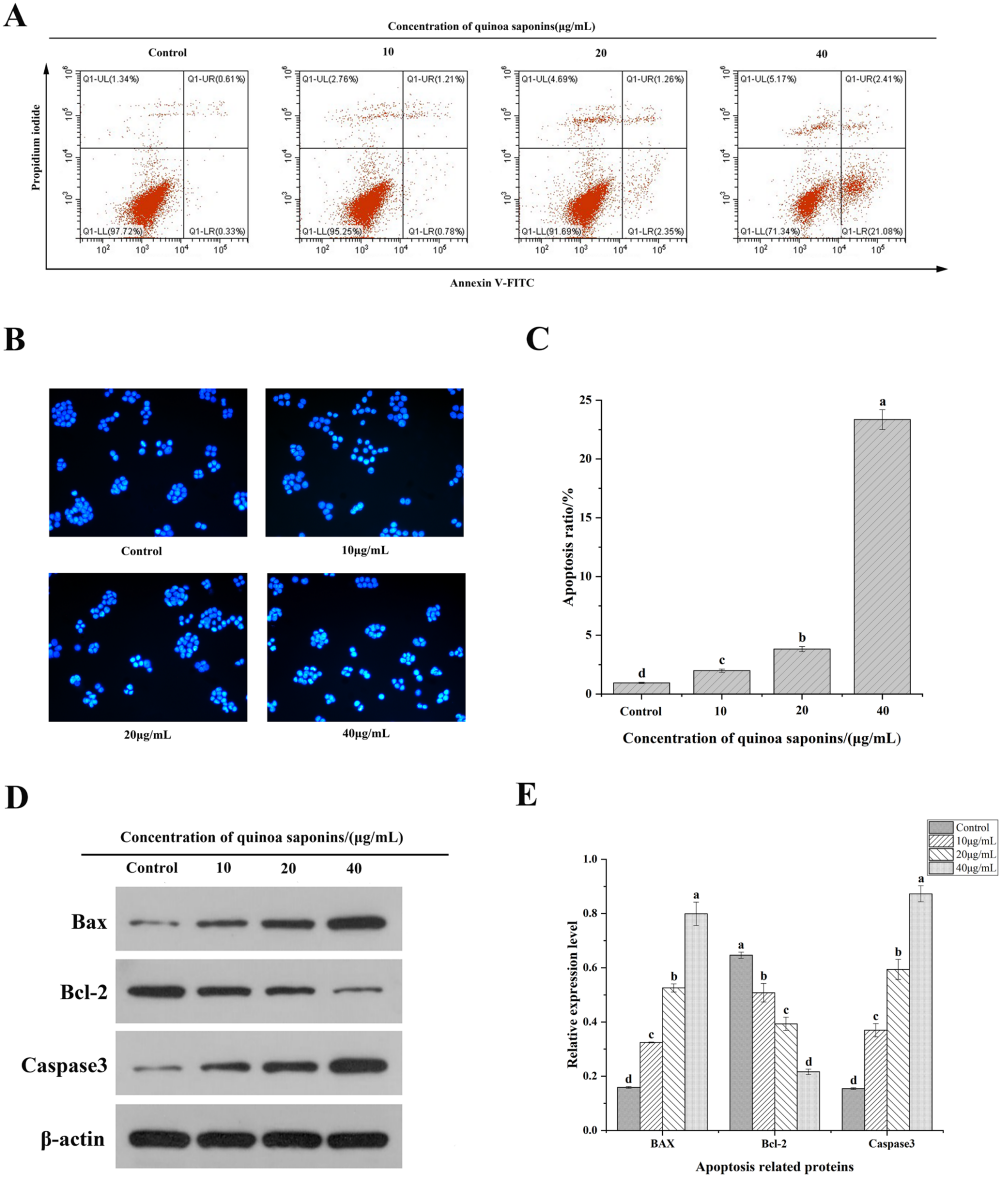
Quinoa saponin‐induced apoptosis of HT‐29 cells. (A) Hoechst 33342 staining was used to observe whether the cells had apoptosis. (B) and (C) The effects of different concentrations of quinoa saponins on apoptosis of HT‐29 cells were detected by flow cytometry. (D) and (E) The expression levels of Bax, Bcl‐2, and Caspase were detected by Western blot. Different letters indicate significant differences, *p* < 0.05.

To determine whether quinoa saponins induced apoptosis in HT‐29 cells, Hoechst 33342 staining was used. Hoechst 33342 stains apoptotic cells bright blue when apoptosis occurs. As shown in Figure 3B, the cells in the control group showed a consistent light blue color, whereas HT‐29 cells treated with quinoa saponins showed varying degrees of bright blue color, indicating the presence of apoptotic bodies. With the increase in concentration of quinoa saponins, this phenomenon showed more obvious. Yao et al. (2020) and Elkady, Hussein, and El‐Assouli (2015) found that the state of cells treated with saponins was consistent with this experiment and led to apoptosis of colon cancer cells. This finding indicated that the treatment with quinoa saponin induced apoptosis in HT‐29 cells.

To determine whether the inhibition of cell growth by quinoa saponins is related to the activation of apoptosis, Annexin V‐FITC/PI staining was used to investigate the effect of quinoa saponins on apoptosis in HT‐29 cells. As shown in Figure 3A,C, after the cells were treated with quinoa saponins, the percentage of live cells decreased significantly, whereas the rate of apoptosis increased. Chen investigated that the effect of paris saponin II on the apoptotic rate of HT‐29 cells was significant in a dose‐dependent manner, which is consistent with the findings of this experiment (Chen et al. 2019). This was also confirmed by Chang's study of the inhibitory impact of total saponins of Rhizoma panacis majoris on apoptosis in colon cancer cells (Chang et al. 2021). The effect of apoptosis was most pronounced after treatment of cells with quinoa saponins (40 μg/mL), which increased the apoptosis rate by 22.55%, compared with that of the control group. The findings revealed that quinoa saponins hastened the apoptosis of HT‐29 cells, resulting in cell growth inhibition. However, the mechanism by which quinoa saponins induce apoptosis in HT‐29 cells is still unclear, and further research will be conducted.

Key regulators of the apoptotic pathway include the antiapoptotic protein Bcl‐2 and the pro‐apoptotic protein Bax (Dadsena, King, and García‐Sáez 2021). As shown in Figure 3D,E, after the cells were treated with quinoa saponins, the expression of Bax and Caspase 3 increased in a dose‐dependent manner, whereas the expression of Bcl‐2 decreased continuously. Li studied the effects of three active monomers of Trillium saponins on colon cancer and reached the same conclusion (Li, Liu, et al. 2015 ). Bax facilitates cell death by modulating the permeability of the outer mitochondrial membrane, whereas Bcl‐2 suppresses apoptosis by reducing Bax activity (Martinou and Youle 2011). In this experiment, the Bcl‐2/Bax ratio continued to decrease, indicating that quinoa saponin induced apoptosis in HT‐29 cells by triggering the mitochondrial‐mediated endogenous apoptosis pathway, affecting cell growth.

### 3.4 Quinoa Saponin‐Induced Autophagy Inhibited the Proliferation of HT‐29 Cells

**FIGURE 4:**
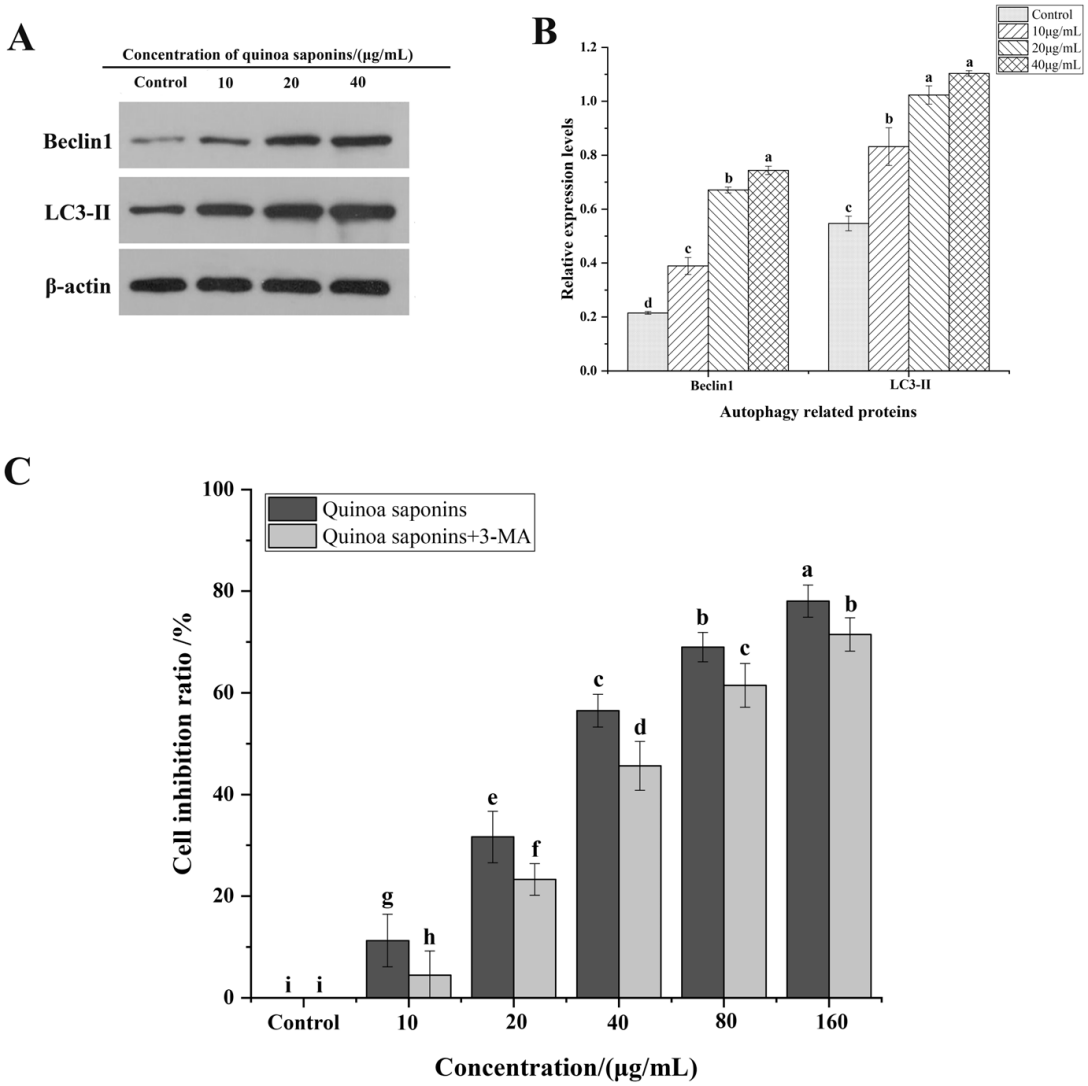
Quinoa saponin‐induced autophagy inhibited the proliferation of HT‐29 cells. (A) and (B) The expression levels of autophagy‐specific proteins LC3‐II and Beclin1 were detected by Western blot to judge whether the cells produced autophagy. (C) HT‐29 cells were treated with autophagy inhibitors, and the effect of quinoa saponin‐induced autophagy on cell proliferation was detected by MTT. Different letters indicate significant differences, *p* < 0.05.

Beclin1 and LC3 are regarded as the primary markers of autophagy ( Li, Zou, et al. 2021). To investigate whether quinoa saponins can induce autophagy in HT‐29 cells, the expression of LC3‐II and Beclin1 in HT‐29 cells was analyzed by Western blotting. As shown in Figure 4A,B, the expression of Beclin1 and LC3‐II was upregulated in a dose‐dependent manner. Gao et al. (2018) found that ophiopogonin B induced autophagy in colon cancer cells and the expression of Beclin1 and LC3‐II was upregulated. Han et al. (2024) also confirmed that autophagy was caused by upregulation of Beclin1 and LC3‐II expression. The results indicated that quinoa saponin induced autophagy in HT‐29 cells.

Autophagy can either enhance cell growth vigor or inhibit cell proliferation. To determine whether the autophagy induced by quinoa saponins has a protective or inhibitory effect on cells, cells were pretreated with an autophagy inhibitor (3‐MA). As shown in Figure 4C, the inhibitory effect of the saponins group pretreated with 3‐MA on the cells was significantly lower than that of the group treated with the same concentration of saponins. When the concentration of quinoa saponins was 40 μg/mL, the cell growth inhibition rate decreased by 10.88% after 3‐MA pretreatment. The results indicated that 3‐MA partially prevented quinoa saponin‐induced cell death. This experiment's findings are in line with Wu's discovery that ginsenoside‐induced autophagy in colon cancer cells inhibited cell proliferation (Wu et al. 2018). It can be inferred that quinoa saponin‐induced autophagy has an inhibitory effect on the growth of HT‐29 cells, and its anticancer activity may be related to the induced autophagy.

### 3.5 Quinoa Saponins Inhibited HT‐29 Cells' Migration In Vitro

**FIGURE 5:**
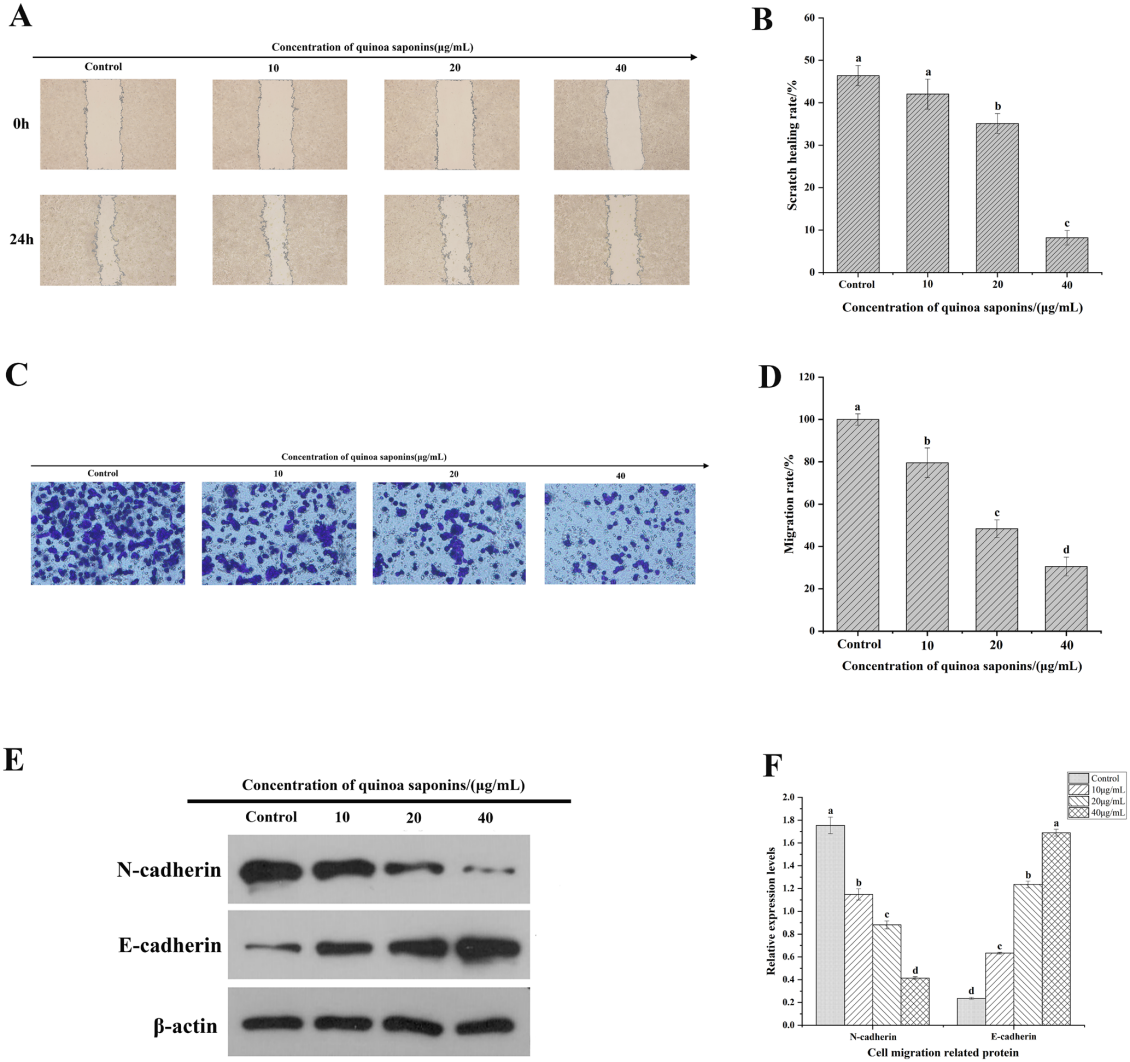
Quinoa saponins inhibited HT‐29 cell migration in vitro. (A) and (B) The wound healing degree of HT‐29 cells treated with different concentrations of quinoa saponins was observed by the scratch test. (C) and (D) The migration ability of HT‐29 cells treated with different concentrations of quinoa saponins was detected by Transwell. (E) and (F) The expression levels of E‐cadherin and N‐cadherin were detected by Western blot. Different letters indicate significant differences, *p* < 0.05.

During tumorigenesis, accelerated migration of cancer cells promotes tumor spread and metastasis (Lambert, Pattabiraman, and Weinberg 2017). The effect of quinoa saponins on the migration ability of HT‐29 cells was detected by the scratch test and Transwell. As shown in Figure 5A,B, the scratch healing rate of the quinoa saponin‐treated group was significantly lower in a dose‐dependent manner than that of the control group. It indicated that high concentrations of quinoa saponins could significantly inhibit the wound healing ability of HT‐29 cells, resulting in the blockage of the migration ability of the cells. As shown in Figure 5C,D, the number of migrated HT‐29 cells was significantly reduced with the increase in the concentration of quinoa saponins. The inhibition of cell migration was more pronounced after treatment with quinoa saponin (40 μg/mL). The cell healing and migration abilities were reduced by 38.21% and 69.48%, respectively, in comparison to those of the control group. This is consistent with Yuan's finding that ginsenoside Rh2 reduced the migratory ability of colon cancer cells in a dose‐dependent manner (Yuan et al. 2020). Fan also obtained the same results (Fan et al. 2015). The findings demonstrated that quinoa saponins dramatically inhibited the migration of HT‐29 cells.

The epithelial‐mesenchymal transition (EMT) is intimately associated with the migration of cancer cells. The expression of N‐cadherin is upregulated, whereas that of E‐cadherin is downregulated, which are its main characteristics (Craene and Berx 2013). As shown in Figure 5E,F, the expression of E‐cadherin increased and the expression of N‐cadherin decreased after treatment with quinoa saponins. The effect was more pronounced after treatment with quinoa saponins (40 μg/mL). Yuan came to the same conclusion that ginsenosides affected the EMT of cancer cells, thus preventing colon cancer metastasis (Yuan et al. 2020). The results indicated that quinoa saponins significantly affected the expression of E‐cadherin and N‐cadherin in HT‐29 cells, thereby inhibiting the occurrence of EMT in HT‐29 cells. This may be the mechanism by which quinoa saponins inhibit the migration of HT‐29 cells.

## 4 Discussion

In recent years, the incidence rate of colon cancer has been increasing. Many studies have confirmed that active ingredients in plants can effectively inhibit the survival of tumor cells and selectively target effective targets of malignant proliferation genes, which has become a new strategy for tumor therapy (Li et al. 2013). The analysis of chemical composition of quinoa has shown that quinoa saponins are the main active substances of quinoa and have anticancer effects. However, there are few reports on the effects of quinoa saponins on HT‐29 cells and their mechanism of action. In this experiment, quinoa saponins were isolated from quinoa seed coat and their effects on colon cancer were investigated. The findings demonstrated that quinoa saponins dramatically decreased the survival of HT‐29 cells.

Cell proliferation is an important part of normal cellular physiological activities. In this experiment, quinoa saponins inhibited the proliferation of HT‐29 cells in a dose‐ and time‐dependent manner. Numerous investigations have verified that the cell cycle plays a significant role in controlling the proliferation of cells. Jaramillo et al. (2016) found that steroidal saponins in wild asparagus caused colon cancer cells to be arrested in the G0/G1 phase, which affected cell proliferation. Gao et al. (2018) found that ophiopogonin B inhibited the proliferation of colon cancer cells by regulating the blockage of the G0/G1 phase. Uncontrolled cell cycle progression is one of the key traits of tumor cells, and therefore, blocking this process is considered a target for cancer therapy. The cell cycle is regulated by a variety of intracellular molecules, among which the expression of cell cycle proteins is temporal and periodic. Cyclin D1 is one of the cell cycle proteins. Downregulation of Cyclin D1 expression can cause cell cycle arrest in the G0/G1 phase and inhibit the proliferation of tumor cells (Cho et al. 2017). Overexpression of Cyclin D1 can cause cells to transition from the G0/G1 cycle to the S phase (Lim and Kaldis 2013). P21 is a CDK inhibitor whose increased expression is associated with the G0/G1‐phase blockade, which can play an inhibitory role in tumor cells by controlling the progression of the cell cycle (Deng et al. 2018). Quinoa saponin induced significant G0/G1 phase arrest in HT‐29 cells by regulating the expression of Cyclin D1 and P21, thereby inhibiting the proliferation of colon cancer cells. In contrast, Yao found that polyphyllin PP9 effectively induced G2/M‐phase arrest in HT‐29 cells (Yao et al. 2020), which is different from the cell cycle blocked in this experiment. This may be because saponins affect the cell cycle distribution by regulating the expression of different proteins in cells, thereby affecting the proliferation of cancer cells.

Apoptosis is the genetically controlled autonomous programmed death of cells. Under normal circumstances, the body clears damaged or diseased cells through apoptosis to maintain its health (Liu et al. 2017). There are two pathways for cell apoptosis: endogenous and exogenous. The endogenous pathway is mainly initiated by DNA damage and metabolic stress at the mitochondrial level (Brentnall et al. 2013). An important feature of endogenous apoptosis is the release of cytochrome C from mitochondria into the cytoplasm, leading to the formation of apoptotic bodies and activation of Caspase 3. Bcl‐2 is an antiapoptotic protein whose high expression may lead to abnormal proliferation of colon cancer cells. The pro‐apoptotic protein Bax is activated and translocated to the outer membrane of the mitochondria, increasing the permeability of the membrane (Dadsena, King, and García‐Sáez 2021). This causes cytochrome C to be released from the mitochondria, which starts the caspase cascade reaction. As downstream products of cytochrome C, caspases play a key role in apoptosis (Siedlecka‐Kroplewska, Wozniak, and Kmiec 2019). Caspase‐9 is a major initiator upstream of the apoptotic cascade effect, and Caspase‐3 is considered to be the most important apoptotic executor among the members of the Caspase family, which enables specific cleavage of a number of key proteins in the cell (Xu et al. 2021). In addition, the decrease in Caspase‐3 may inhibit apoptosis of tumor cells, thus promoting tumorigenesis. In Zhang's study, the expression of Caspase‐3 was decreased and cell proliferation was significantly increased during lung carcinogenesis in rats (Zhang et al. 2019). Hong discovered that the anticancer effects of ginsenoside Rg3 might cause cancer cells to undergo apoptosis by triggering the caspase‐3 and caspase‐9 pathways (Hong et al. 2020). This finding is important for a deeper understanding of the mechanism of apoptosis in tumor cells and for the development of new anticancer drugs. To ascertain whether the suppression of cell proliferation brought on by quinoa saponins is connected to the activation of cell apoptosis, this study confirmed through morphology that quinoa saponin induced cell apoptosis. Western blotting showed that the relative expression ratio of Bcl‐2/Bax was continuously decreasing and activated Caspase‐3 protein. These results suggested that quinoa saponin induced apoptosis in HT‐29 cells by triggering the mitochondria‐mediated endogenous apoptotic pathway, which affects cell growth.

Autophagy is also a type of programmed cell death, in which cells can degrade and digest damaged organelles, misfolded proteins, and invading pathogens through the lysosome‐mediated process of autophagy (Jung, Jeong, and Yu 2020). Beclin‐1 and LC3 proteins are major markers of autophagy (Du et al. 2017). Beclin‐1 gene is a specific autophagy‐related gene, and the Beclin‐1 protein encoded by it is considered to be a tumor suppressor protein, which is able to regulate autophagy process, participate in the nucleation stage, and induce the aggregation of autophagy‐associated proteins (Kang et al. 2011). LC3 is regarded as an important player in monitoring cellular autophagy, and the cytoplasmic LC3 (LC3‐I) will be converted to active LC3‐II and bound to the autophagosome membrane, so the number of autophagosomes can be reflected by detecting the expression of LC3‐II. Gao found that Beclin1 and LC3‐II were elevated after treatment with Ophiopogon saponin B, which indicated that Ophiopogon saponin B induced autophagy in cells (Gao et al. 2018). In this experiment, the expression of Beclin1 and LC3‐II in the cells increased significantly with the increase in the concentration of quinoa saponins, so it was determined that the treatment of quinoa saponin induced autophagy in HT‐29 cells. Autophagy disorders are closely related to tumor development, and the process of cellular autophagy can both enhance the growth vitality of tumor cells and inhibit the proliferative effects of tumor cells. Therefore, the combined application of autophagy promoters or autophagy inhibitors with chemotherapeutic drugs has become a new direction in antitumor therapy. This study confirmed that quinoa saponin induced autophagy in cells and had an inhibitory effect on the proliferation of HT‐29 cells. This is consistent with Wu's findings on the effects of ginsenosides on colon cancer cells (Wu et al. 2018). However, Han found that autophagy induced by platycodin D in HT‐29 cells could protect the cells (Han et al. 2024). Therefore, not all autophagy is a single process that promotes cell death or protects cells, and the specific mechanisms of autophagy need to be further investigated.

Metastasis is the main cause of death in most patients with malignant tumors, so inhibition of metastasis is a priority in clinical treatment of tumors. Many studies have confirmed that EMT is a critical step in tumor metastasis. EMT is a key mechanism for early acquisition of migration ability in malignant tumors derived from epithelial cells (Cao et al. 2015). It has been found that EMT allows epithelial cells to be transformed into cells with mesenchymal characteristics, and the polarity of the cells gradually disappears, making cell migration stronger. Therefore, inhibiting or blocking EMT may be an effective means to limit tumor cell metastasis. E‐cadherin and N‐cadherin, as marker proteins in the EMT process, play a crucial role in mediating cell adhesion, inhibiting tumor invasion and metastasis (Brabletz et al. 2018). The downregulation of E‐cadherin expression is one of the key links in inducing EMT, whereas the upregulation of N‐cadherin expression is involved in gene transcriptional regulation (Na et al. 2020; Yan et al. 2021). Xia investigated that the saponins of patrinia villosa exerted antitumor metastasis by inhibiting EMT in colon cancer cells, and the mechanism may be through regulating the expression of E‐cadherin and N‐cadherin (Xia et al. 2018). Li found that the ginsenoside Rg3 could effectively inhibit the metastasis of colon cancer cells. By promoting the expression of E‐cadherin, Rg3 could inhibit the EMT process of colon cancer cells (Li, Liu, et al. 2021 ). This study discovered that quinoa saponins might limit the EMT process by upregulating the expression of E‐cadherin and downregulating that of N‐cadherin, thereby greatly inhibiting the migratory ability of HT‐29 cells. This may be the mechanism by which quinoa saponins prevent migration of HT‐29 cells.

## 5 Conclusion

In conclusion, quinoa saponins significantly inhibited the proliferation and migration of HT‐29 cells, induced cell cycle arrest, and mediated apoptosis through the mitochondrial apoptotic pathway. Furthermore, it was found that autophagy induced by quinoa saponins in HT‐29 cells promotes cell death. The mechanism of action of quinoa saponins on HT‐29 cells was preliminarily investigated. However, there are also shortcomings in this study, such as the lack of in‐depth research on relevant signaling pathways and drug targets, and the lack of corresponding animal experiments. In the later study, we will further explore its specific mechanism, establish animal models for in vivo evaluation of drug efficacy, and provide more reference basis for the later in‐depth development and application.

## Author Contributions

**Haijun Shang:** conceptualization (equal), data curation (equal), formal analysis (equal), investigation (equal), methodology (equal), software (equal), validation (equal), writing – original draft (lead). **Jinwei Sun:** data curation (equal), investigation (equal), methodology (equal), software (equal), validation (equal), writing – review and editing (equal). **Zhi Zheng:** formal analysis (equal), funding acquisition (supporting), investigation (equal), methodology (equal), supervision (equal), validation (equal), writing – review and editing (equal). **Shujun Sun:** data curation (equal), formal analysis (equal), software (equal), writing – review and editing (equal). **Xiaoming Yan:** conceptualization (equal), funding acquisition (lead), project administration (lead), resources (lead), supervision (equal), writing – review and editing (equal).

## Conflicts of Interest

The authors declare no conflicts of interest.

## Data Availability

The datasets generated for this study are available on request from the corresponding author.
